# Prognostic Nomogram for Patients with Nasopharyngeal Carcinoma after Intensity-Modulated Radiotherapy

**DOI:** 10.1371/journal.pone.0134491

**Published:** 2015-08-06

**Authors:** Shixiu Wu, Bing Xia, Fei Han, Ruifei Xie, Tao Song, Lixia Lu, Wei Yu, Xiaowu Deng, Qiancheng He, Cong Zhao, Conghua Xie

**Affiliations:** 1 Department of Radiation and Medical Oncology, Zhongnan Hospital, Wuhan University, Wuhan, Hubei, P. R. China; 2 Hubei Cancer Clinical Study Center, Wuhan University, Wuhan, Hubei, P. R. China; 3 Department of Radiation Oncology, Hangzhou Cancer Hospital, Hangzhou, Zhejiang, P. R. China; 4 State Key Laboratory of Oncology in Southern China, Guangzhou, P. R. China; 5 Department of Radiation Oncology, Sun Yat-Sen University Cancer Center, Guangzhou, P. R. China; 6 Collaborative Innovation Center of Cancer Medicine, Guangzhou, P. R. China; 7 Department of Radiation Oncology, Wenzhou Medical College Cancer Center, Wenzhou, Zhejiang, P. R. China; National Cancer Centre, SINGAPORE

## Abstract

This study was aimed to define possible predictors of overall survival in nasopharyngeal carcinoma (NPC). Patients were treated with intensity-modulated radiation therapy (IMRT), to establish an effective prognostic nomogram that could provide individualized predictions of treatment outcome in this setting. We reviewed the records of 533 patients with non-metastatic NPC who underwent IMRT with or without concurrent chemotherapy at the Department of Radiation Oncology of Sun Yat-Sen University from 2002 to 2009; none of these patients received induction or adjuvant chemotherapy. These data sets were used to construct a nomogram based on Cox regression. Nomogram performance was determined via a concordance index (C-index) and a calibration curve which was compared with the TNM staging system for NPC. The results were validated in an external cohort of 442 patients from the Department of Radiation Oncology of Wenzhou Medical College who were treated during the same period. Results showed that the greatest influence on survival were primary gross tumor volume, age, tumor stage and nodal stage (2002 Union for International Cancer Control [UICC] staging system), which were selected into the nomogram. The C-index of the nomogram for predicting survival was 0.748 (95%CI, 0.704–0.785), which was statistically higher than that of TNM staging system (0.684, P<0.001). The calibration curve exhibited agreement between nomogram-predicted and the actual observed probabilities for overall survival. In the validation cohort, the nomogram discrimination was superior to the TNM staging system (C-index: 0.768 vs 0.721; P = 0.026). In conclusion, the nomogram proposed in this study resulted in more-accurate prognostic prediction for patients with NPC after IMRT and compared favorably to the TNM staging system; this individualized information will aid in patient counseling and may be used for de-escalation trials in the future.

## Introduction

Nasopharyngeal carcinoma (NPC) is a leading cancer in Southeast Asia, the Arctic and the Middle East/North Africa although it is uncommon worldwide. Globally, NPC resulted in 65,000 deaths in 2010, an increase from 45,000 in 1990[1]. Radiationtherapy (RT) has been the mainstay of curative treatment for NPC for decades. Currently, Intensity-Modulated Radiotherapy (IMRT) is the preferred standard of care in non-metastatic NPC, which significantly improves coverage of the target tumor and spares normal structures, leading to increased local-regional control and reduced RT-related sequelae[2–6].With IMRT, 3-year local and regional control rates which exceed 90% have been documented; However, distant metastases (DM) have become the main treatment failure, with 3-year DM rates of 20%-34% [2,4,7–11]. Although the addition of induction or adjuvant chemotherapy in NPC is a reasonable option for patients at high risk for developing DM, the survival benefits are unclear. Considerable toxicities related to concurrent chemoradiotherapy potentially reduce patient’s tolerability, leading to a compromised systemic therapy, which should be considered as a confusing factor. Meanwhile, administration of systemic therapy in a poorly selected population easily leads to negative results of trials [12–15].

Accurate prognostic models for individualized predictions will be propitious to identify and stratify patients for receiving systemic chemotherapy on or off a clinical trial in NPC. The Union for International Cancer Control (UICC) TMN staging provides useful estimates for recurrence risk and survival outcomes, significant variation within each prognostic group has been observed. Variation due to the heterogeneity of tumor biology and patient characteristics, indicate that more relevant variables should be integrated to improve the estimates of patient outcomes. The currently used staging system was mainly derived from the data based on two-dimensional radiation techniques therefore its suitability for patients treated with IMRT remains unclear.

There are many studies in which nomogram has been developed and been demonstrated as a useful tool for providing prognostic information in various cancer types [16–21].The purpose of this study was to define possible predictors of overall survival in NPC patients treated with IMRT, and to establish an effective prognostic nomogram which could provide an individualized prediction for the treatment outcome. In order to reduce the impact of systemic chemotherapy heterogeneity on the outcome, only those with concurrent chemotherapy of cisplatin alone were included in this study.

## Methods

### Study Population

This retrospective study was conducted in patients treated with IMRT between September 2002 and December 2009 at two NPC-endemic areas in China (Department of Radiation Oncology, Sun Yat-Sen University, training cohort; Department of Radiation Oncology, Wenzhou Medical College, validation cohort). To collect data for the nomogram development, an electronic survey form was designed to select patients and gather relevant information before study initiation. This analysis was approved by the Institutional Review Boards, with both participating sites providing the necessary institutional data use agreements (Sun Yat-Sen University and Wenzhou Medical College). Patients’ records were anonymized and de-identified prior to analysis. The following inclusion criteria were used for this study: histopathologically proven NPC, prior administration of IMRT with curative intention, no history of previous anticancer therapy and no history of other malignancies. The exclusion criteria were as follows: evidence of DM at diagnosis, prior administration of induction/adjuvant chemotherapy, prior administration of non-cisplatin concurrent chemotherapy and incomplete information for the follow-up.

Pre-treatment staging procedures consistently included clinical history, physical examination, biochemical test, magnetic resonance imaging (MRI) or computer tomography (CT) scan of the head and neck, chest radiography, and ultrasonography of the abdominal region. Additional investigations were performed if indicated. Stage data was retrieved from the original chart reports and was unified to 2002 UICC staging system; in case of incomplete stage information, original images were retrieved and reviewed by our study radiation oncologists.

The techniques of planning and delivery of IMRT used in the two cohorts were described previously [2,11]. The prescribed dose was 68–70 Gy/28-30 fractions to the gross target volume, 56–60 Gy/28-30 fractions to the high-risk clinical target volume and 45–54 Gy/23-30 fractions to the low-risk clinical target volume. At the beginning the use of IMRT and concurrent chemotherapy was not mandatory for local advanced NPC in both cancer center guidelines, provided that good local-regional control with IMRT alone and high toxicity incidence rates when RT was delivered with concurrent chemotherapy. With more and more evidence supported using concurrent chemotherapy for advanced NPC and acceptable toxicity profiles reported, this strategy was accepted gradually in clinical practice but often with a reduced dose of chemotherapy. Generally, concurrent chemotherapy was delivered with cisplatin alone (80 mg/m^2^/d on days 1 and 22 or 25 mg/m^2^/d weekly).

### Statistical Analysis

The objective of this study was to define possible predictors of overall survival (OS) in NPC patients treated with IMRT, and to establish an effective prognostic nomogram. OS was estimated using the Kaplan-Meier method, and comparisons were assessed using the log-rank test. A multivariate Cox proportional hazards model was used to analyze the risk factors associated with OS. The variables used to construct the nomogram were selected a priori based on previous research and data availability; they included the gender, age, clinical tumor (T) stage, clinical nodal (N) stage, primary gross tumor volume (GTV) and administration of concurrent chemotherapy (yes/no) [22].The final model selection was performed using a backward step-down selection process with the Akaike information criterion [23] and the *rms* package in R version 3.0.1.

Nomogram performance was measured using a concordance index (C-index), which can be applied to continuous outcome and censored data. The nomogramcalibrationwas assessed by plotting the observed rates against the nomogram-predicted probabilities. Bootstrap analyses with 1,000 resamples were used for these activities. The nomogram and UICC staging system were compared by evaluating the C-index. During the external validation of the nomogram, the survival probability of each patient in the validation cohort was calculated according to the established nomogram; these values were used to plot receiver operating characteristic (ROC) curves by SPSS 19.0 for Windows. In general, areas under the curve (AUC) of 0.7 to 0.8 represent reasonable discrimination [24].

## Results

### Nomogram Development

The clinical and treatment characteristics of patients in the training cohort (*N* = 533) are presented in Table 1. In total, 371 patients were classified as stage III/IV, of which 280 (76%) patients were administered concurrent chemotherapy. The median follow-up time was 84.2 months (range 4.2–141.8 months) for all patients; 382 (72%) patients had at least 5 years of follow-up time. Of the 533 patients, 35 (7%) and 84 (16%) patients experienced local-regional recurrence and DM, respectively. In addition, 118 (22%) patients died at last follow-up. The 3- and 5-year OS rates were 89.1% (95%CI 86.6–91.6%) and 81.7% (95%CI 78.4–85.0%), respectively.

**Table 1:** Clinical and treatment characteristics of NPC patients.

Characteristics	primary cohort (*N* = 533)		validation cohort (*N* = 442)	
	No. of patients	%	No. of patients	%
**Age(years)**				
Median (range)	43(13–78)		53(24–82)	
Age≤median	280	53	239	54
Age<median	253	47	203	46
**Sex**				
Male	411	77	294	67
Female	122	23	148	33
**Stage**				
I	33	6	41	9
II	129	24	130	29
III	274	52	171	39
IV	97	18	100	23
**T stage**				
1	49	9	107	24
2	186	35	140	32
3	215	40	111	25
4	83	16	84	19
**N stage**				
0	159	30	123	28
1	196	37	177	40
2	163	30	113	26
3	15	3	29	6
**Gross tumor volume(mm3)**				
Median (range)	22.4 (1.1–205.5)		25.7(0.7–182.7)	
GTV≤median	267	50	222	50
GTV<median	266	50	220	50
**concurrent chemotherapy**				
No	212	40	398	90
Yes	321	60	44	10

**Fig 1:**
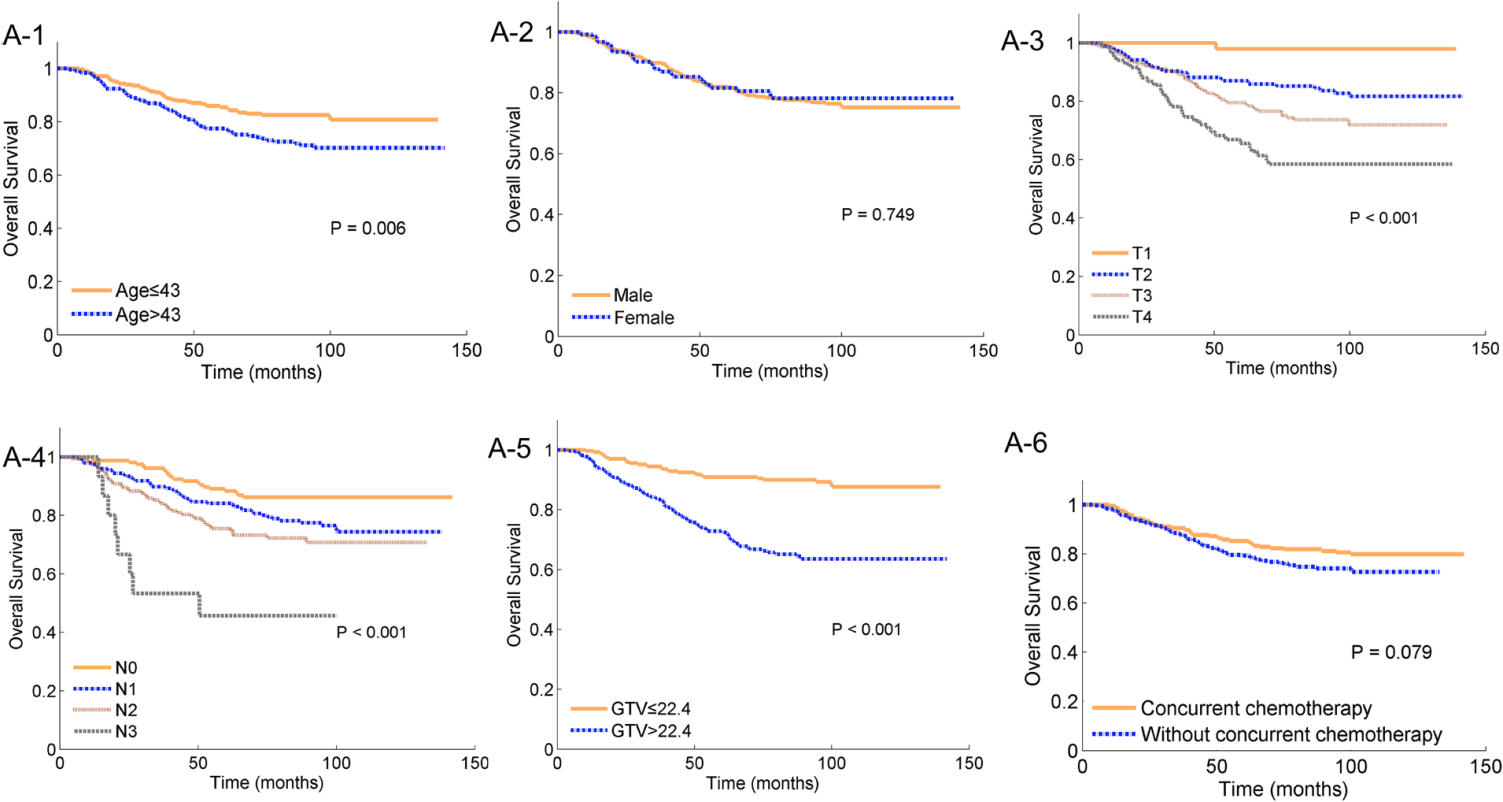
Kaplan-Meier survival plots of overall survival stratified according to age (≤43 or >43 years), gender (male or female), T stage (1, 2, 3 or 4), N stage (0, 1, 2 or 3), gross tumor volume (GTV, ≤22.4 or >22.4 mm^3^) and concurrent chemotherapy (yes or no) for nasopharyngeal carcinoma patients treated with intensity-modulated radiation therapy in the training cohort (*N* = 533). The log-rank test results for each comparison are provided.

**Fig 2:**
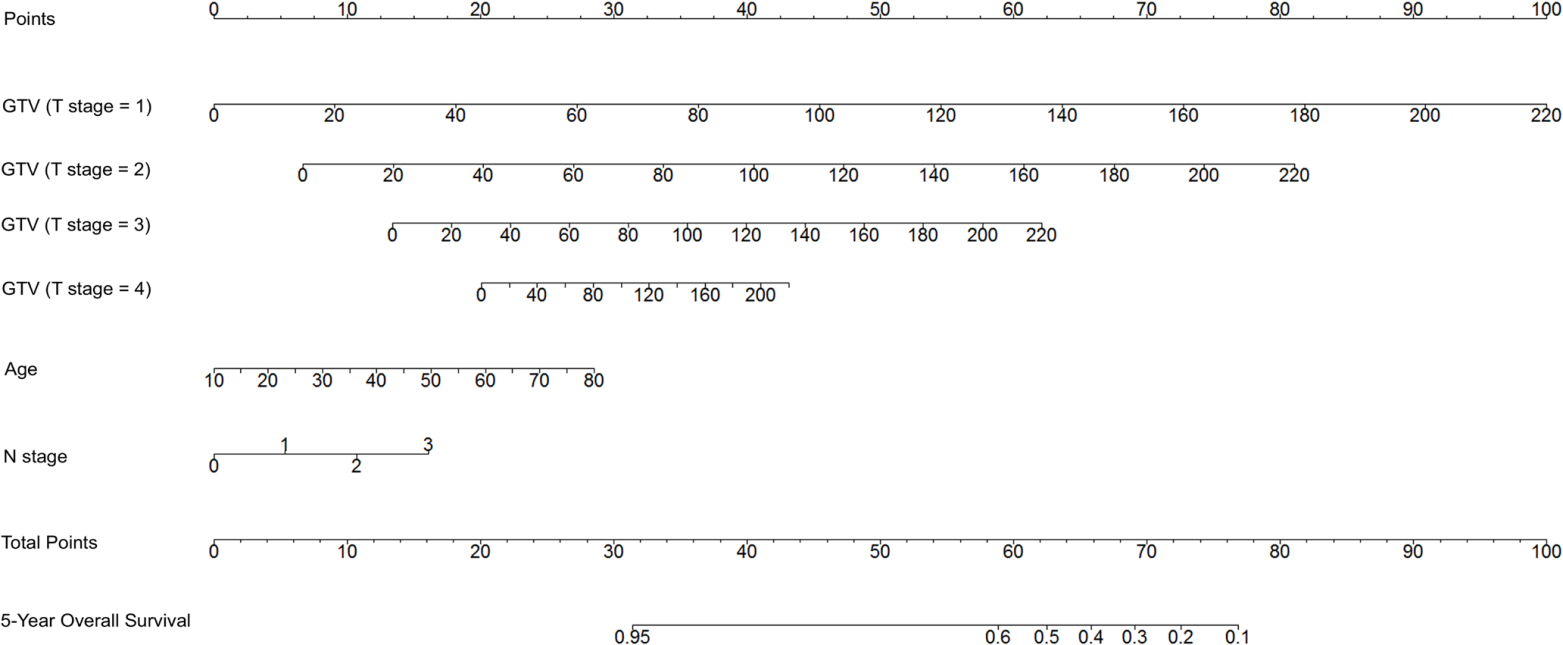
The nomogram developed for the 5-year prediction of overall survival. Point scores for gross tumor volume (GTV) were identified based on the T stage. To estimate risk, points for each variable were calculated by drawing a straight line from a patient’s variable value to the axis labeled “Points.” The score sum is converted to a probability in the lowest axis.

**Fig 3:**
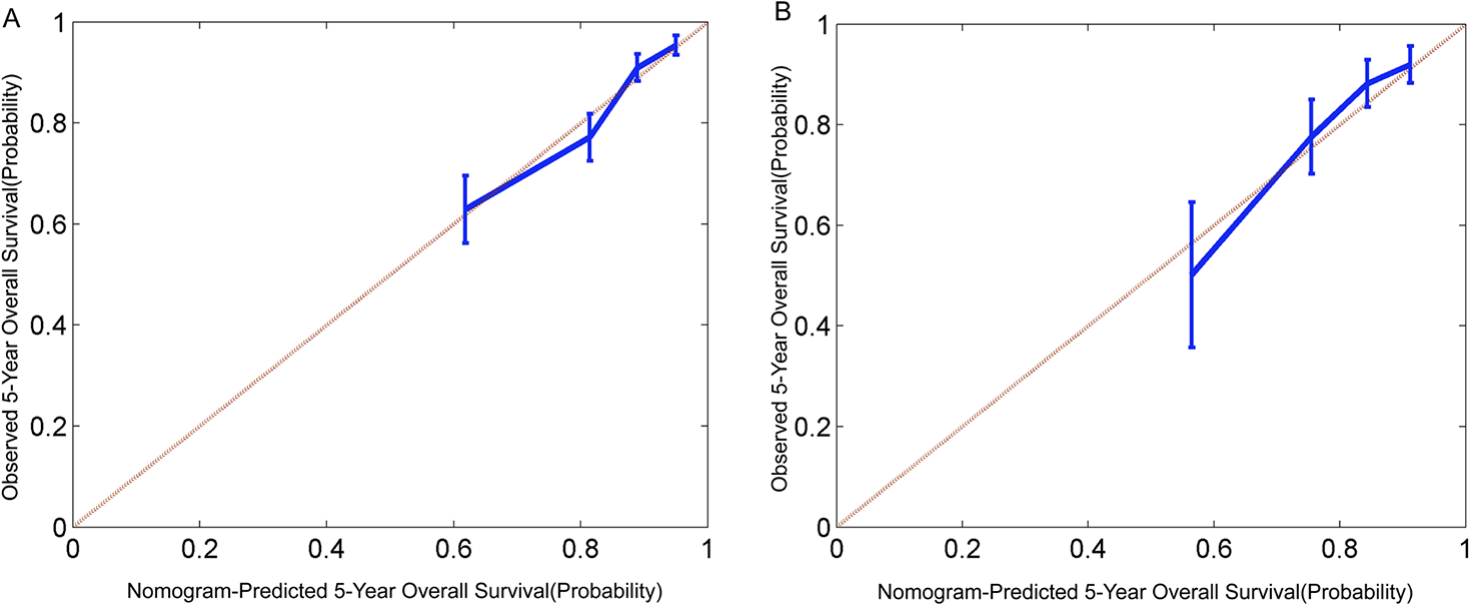
(A) Calibration curves for 5-year probabilities of overall survival in the training cohort of 533 patients and (B) the validation cohort of 442 patients. Patients were grouped by quartiles of predicted risk. Nomogram-predicted probability is plotted on the x-axis; observed probability is plotted on the y-axis (Kaplan-Meier estimates). Vertical bars = 95% confidence index.

Age, T stage, N stage and GTV were significantly associated with OS in Kaplan-Meier analyses (Fig 1); the use of concurrent chemotherapy exhibited a tread toward improved OS in the Kaplan-Meier analysis but had no significant effect on OS in multivariate analysis (Table 2). Given that patients with high T stages may exhibit a large GTV, the interaction between the T stage and GTV was analyzed using a Cox proportional hazards model; the results revealed a positive effect (P = 0.009). Therefore, the T stage was subcategorized according to 4 levels, and the interactions between the T stage and GTV are presented as four scale bars in the development of the nomogram for OS (Fig 2). Internal validation using bootstrapping revealed a C-index of 0.748 (95%CI 0.704–0.785), which was significantly increased compared with that of the 2002 UICC staging system (0.684; 95%CI 0.640–0.728, P<0.001). The 5-year probability of OS for the nomogram exhibited good model calibration with high correlations with the nomogram-predicted and observed probabilities of OS (Fig 3A).

**Table 2:** Multivariate analyses of overall survival with Cox proportional hazards model for patients with nasopharyngeal carcinoma in the training cohort (*N* = 533).

	Multivariate analysis	
	*P* value	HR(95%CI)
Age	<0.001	1.034(1.018–1.051)
T stage	0.003	1.812(1.227–2.677)
N stage	<0.001	1.595(1.260–2.019)
GTV	<0.001	1.049(1.023–1.076)
T*GTV	0.009	0.990(0.983–0.998)
Concurrent chemotherapy	0.324	0.816(0.545–1.222)

Abbreviations: T stage, clinical tumor stage; N stage, clinical nodal stage; GTV, gross tumor volume; HR, hazard ratio.

### External Validation

The external validation cohort consisted of 442 patients (Table 1). Overall, patients with stage III/IV disease were less likely to be administered concurrent chemotherapy in the validation cohort (13%). The median follow-up time was 30.5 months (range 3.1–119.6 months) for all patients; 77(17%) patients had at least 5-year of follow-up time. Of the 442 patients with NPC, 24 (5%) and 54(12%) patients experienced local-regional recurrence and DM, respectively. In addition, 64 (15%) patients died at last follow-up. The 3- and 5-year OS rates were 85.3% (95%CI 81.4–89.2%) and 77.9% (95%CI 72.4–83.4%), respectively.

ROC curves were generated to assess the discrimination of the nomogram in the external population. The overall predictive accuracy of the nomogram for 5-year OS as measured by the AUC was 0.784 (95%CI 0.704–0.865), which was significantly increased compared with that of the 2002 UICC staging system (0.721; 95%CI 0.622–0.820, P = 0.026). The actual 5-year OS was also plotted against the calculated predicted 5-year probabilities of OS for each patient; this alignment exhibited good calibration for the events (Fig 3B).

## Discussion

In this study, we evaluated several prognostic factors of NPC in patients treated with IMRT, and then developed an OS prediction model that can provide individual estimations of treatment outcome in this population, which outperformed the conventional risk model (TNM staging system). An external cohort was used to validate the developed nomogram, revealing a good ability to discriminate OS outcomes. The nomogram developed here does not specifically provide a treatment decision; it simply provides a means to evaluate individual patient outcomes after IMRT.

The current staging system used for NPC is the seventh edition of the UICC/AJCC TNM staging system [25], an obvious limitation of which, is that it excludes the important prognostic factor of tumor burden. Recently published data demonstrated that NPC primary tumor volume is an independent prognostic indicator for treatment outcome in patients treated with IMRT; these studies suggested that the incorporation of GTV into the NPC clinical staging system could provide more information to adjust the treatment strategy [26–29]. In this study, we also noted that large tumors were significantly associated with treatment outcomes and that the incorporation of tumor volume into the TMN staging system significantly improved the ability to identify patients with a poor prognosis. A larger tumor volume often signifies an increased number of clonogenic tumor cells as well as radiotherapy resistance associated with tumor hypoxia; in contrast, the clinical T stage appears more likely to signify the capacity of the tumor to infiltrate the surrounding tissue [30]. These two factors represent different biological characteristics of the tumor. Therefore, the T stage in combination with the tumor volume can better predict OS; both of these factors were incorporated in the final nomogram. As presented in Fig 1, GTV exhibited a greater effect in the T1 stage compared with the T4 stage, indicating that the prognosis of patients with a low T stage was mainly dependent on the tumor volume.

A meta-analysis of chemotherapy in NPC studies, including eight randomized clinical trials, indicated that the addition of chemotherapy improved the absolute OS benefit by 6% in five years [12]. Neither induction nor adjuvant chemotherapy improved OS compared with RT alone; the reduced risk of death was mainly attributed to the administration of concurrent chemotherapy by the mechanism of radiosensitization. With the emergence of new RT techniques (IMRT) and more advanced imaging equipment, high local-regional control has been obtained; thus, the value of concurrent chemotherapy still faces substantial uncertainty and challenges. In the Hong Kong study NPC 9901 [31], patients with regionally advanced NPC in the control arm were administered RT alone (half of the patients were administered 3-DCRT) and achieved a 3-year OS rate of 78%. This result was comparable to the concurrent chemotherapy arm, indicating that the survival benefit from chemotherapy confirmed in a two-dimensional era could be considered as compensation for poor RT delivery. In this study, no positive correlation between concurrent chemotherapy and OS was observed. The chemotherapy doses employed in this study appeared to be lower than standard cisplatin doses used in other phase III clinical trials in NPC [31]. In china, such an approach is often used in clinical practice, with the concern of patient’s physical constitution and compliance to chemoradiotherapy in Eastern population. The lower dose of cisplatin utilized in this study should also be considered as a potential confusing factor for the negative correlation between concurrent chemotherapy and OS. The role of concurrent CT in the era of IMRT requires further testing in the future.

This study was performed using retrospective data and treatment was not assigned in a randomized fashion. To reduce the impact of various regimens and cycles of introduction or adjuvant chemotherapy on OS predictions, only patients administered concurrent cisplatin alone chemotherapy were included in this study. The 3-year OS rates were 89.1% and 85.3% for the training cohort and validation cohort, respectively; these results are comparable to the results of 83–94% reported in a recent series using IMRT with or without induction or adjuvant chemotherapy [3,4,8,9]. Moreover, only 13% of the patients with stage III/IV disease were administered concurrent chemotherapy in the validation cohort however, this regimen did not lead to inferior local control or a poor prognosis. This finding indicated that the positive outcomes of patients exclusively treated with IMRT maybe attributed to more modern and aggressive radiation techniques, which potentially negated the benefit from concurrent chemotherapy.

NPC populations in endemic areas exhibit different racial compositions, histological subtypes and other potential etiological factors compared with NPC populations in the West. SEER data analysis indicates that racial differences exist among NPC patients in the U.S. and that Asians exhibit the best 5-year survival rates after stratification according to stage and histologic type [32]. Non-keratinizing type tumors predominate the NPC population in southern China, and these lesions are generally associated with Epstein–Barr virus (EBV) positivity and a favorable prognosis [33].Therefore, the generalizability of the nomogram developed in this study is limited; it can be used for patient-clinician communication and therapeutic advice in Asian endemic areas. Whether it can be applied to Western populations and patients with keratinizing type tumors remains to be determined.

The strength of the nomogram development included the number of patients involved, the length of follow-up and the 5-year results comparable to contemporary series. A limitation of the study was that T and N stage of 6th instead of 7th UICC staging system were used to construct the nomogram. Main changes in the new staging system are: 1) T2a lesions are now designated as T1; 2) retropharyngeal lymph nodes are considered N1. In our nomogram, T1 and T2 axis had a similar scale; N0 and N1 had a close point. Such changes of T and N staging shifting may cause slight variations when converting to total points and then the probability of 5-year survival rate. Introducing molecular markers of tumor biology in NPC may further enhance the performance of the nomogram. Plasma/serum EBV DNA level was found to be useful in the clinical management of NPC patients [34] however, several problems related to quantifying the EBV DNA load need to be resolved before clinical application, including the optimal detection method, the right sampling time and the reasonable cut-off value.

In conclusion, the nomogram proposed in this study accurately predicted the prognosis for NPC patients treated with IMRT with or without concurrent chemotherapy; this individualized information will aid in patient counseling and may be used for de-escalation trials in the future. Additional studies are required to determine whether the nomogram can be applied to other patient groups.

## References

[1] Xu ZJ, Zheng RS, Zhang SW, Zou XN, Chen WQ. Nasopharyngeal carcinoma incidence and mortality in China in 2009. Chin J Cancer. 2013;32(8):453–60. 10.5732/cjc.013.10118 23863562 PMC3845582

[2] Xiao WW, Huang SM, Han F, Wu SX, Lu LX, Lin CG, et al Local control, survival, and late toxicities of locally advanced nasopharyngeal carcinoma treated by simultaneous modulated accelerated radiotherapy combined with cisplatin concurrent chemotherapy: long-term results of a phase 2 study. Cancer. 2011;117(9):1874–83. 10.1002/cncr.25754 . 21509764

[3] Kam MK, Teo PM, Chau RM, Cheung KY, Choi PH, Kwan WH, et al Treatment of nasopharyngeal carcinoma with intensity-modulated radiotherapy: the Hong Kong experience. International journal of radiation oncology, biology, physics. 2004;60(5):1440–50. 10.1016/j.ijrobp.2004.05.022 . 15590175

[4] Lin S, Pan J, Han L, Zhang X, Liao X, Lu JJ. Nasopharyngeal carcinoma treated with reduced-volume intensity-modulated radiation therapy: report on the 3-year outcome of a prospective series. International journal of radiation oncology, biology, physics. 2009;75(4):1071–8. 10.1016/j.ijrobp.2008.12.015 . 19362784

[5] Kam MK, Leung SF, Zee B, Chau RM, Suen JJ, Mo F, et al Prospective randomized study of intensity-modulated radiotherapy on salivary gland function in early-stage nasopharyngeal carcinoma patients. Journal of clinical oncology: official journal of the American Society of Clinical Oncology. 2007;25(31):4873–9. 10.1200/JCO.2007.11.5501 . 17971582

[6] Pow EH, Kwong DL, McMillan AS, Wong MC, Sham JS, Leung LH, et al Xerostomia and quality of life after intensity-modulated radiotherapy vs. conventional radiotherapy for early-stage nasopharyngeal carcinoma: initial report on a randomized controlled clinical trial. International journal of radiation oncology, biology, physics. 2006;66(4):981–91. 10.1016/j.ijrobp.2006.06.013 . 17145528

[7] Lee N, Xia P, Quivey JM, Sultanem K, Poon I, Akazawa C, et al Intensity-modulated radiotherapy in the treatment of nasopharyngeal carcinoma: an update of the UCSF experience. International journal of radiation oncology, biology, physics. 2002;53(1):12–22. . 12007936 10.1016/s0360-3016(02)02724-4

[8] Tham IW, Hee SW, Yeo RM, Salleh PB, Lee J, Tan TW, et al Treatment of nasopharyngeal carcinoma using intensity-modulated radiotherapy-the national cancer centre singapore experience. International journal of radiation oncology, biology, physics. 2009;75(5):1481–6. 10.1016/j.ijrobp.2009.01.018 . 19386431

[9] Lee N, Harris J, Garden AS, Straube W, Glisson B, Xia P, et al Intensity-modulated radiation therapy with or without chemotherapy for nasopharyngeal carcinoma: radiation therapy oncology group phase II trial 0225. Journal of clinical oncology: official journal of the American Society of Clinical Oncology. 2009;27(22):3684–90. 10.1200/JCO.2008.19.9109 19564532 PMC2720082

[10] Ng WT, Lee MC, Hung WM, Choi CW, Lee KC, Chan OS, et al Clinical outcomes and patterns of failure after intensity-modulated radiotherapy for nasopharyngeal carcinoma. International journal of radiation oncology, biology, physics. 2011;79(2):420–8. 10.1016/j.ijrobp.2009.11.024 . 20452132

[11] Wu S, Xie C, Jin X, Zhang P. Simultaneous modulated accelerated radiation therapy in the treatment of nasopharyngeal cancer: a local center’s experience. International Journal of Radiation Oncology* Biology* Physics. 2006;66(4):S40–S6.

[12] Baujat B, Audry H, Bourhis J, Chan AT, Onat H, Chua DT, et al Chemotherapy in locally advanced nasopharyngeal carcinoma: an individual patient data meta-analysis of eight randomized trials and 1753 patients. International journal of radiation oncology, biology, physics. 2006;64(1):47–56. 10.1016/j.ijrobp.2005.06.037 . 16377415

[13] Langendijk JA, Leemans CR, Buter J, Berkhof J, Slotman BJ. The additional value of chemotherapy to radiotherapy in locally advanced nasopharyngeal carcinoma: a meta-analysis of the published literature. Journal of clinical oncology: official journal of the American Society of Clinical Oncology. 2004;22(22):4604–12. 10.1200/JCO.2004.10.074 . 15542811

[14] Ma J, Mai HQ, Hong MH, Min HQ, Mao ZD, Cui NJ, et al Results of a prospective randomized trial comparing neoadjuvant chemotherapy plus radiotherapy with radiotherapy alone in patients with locoregionally advanced nasopharyngeal carcinoma. Journal of clinical oncology: official journal of the American Society of Clinical Oncology. 2001;19(5):1350–7. . 11230478 10.1200/JCO.2001.19.5.1350

[15] Kwong DL, Sham JS, Au GK, Chua DT, Kwong PW, Cheng AC, et al Concurrent and adjuvant chemotherapy for nasopharyngeal carcinoma: a factorial study. Journal of clinical oncology: official journal of the American Society of Clinical Oncology. 2004;22(13):2643–53. 10.1200/JCO.2004.05.173 . 15226332

[16] Abu-Rustum NR, Zhou Q, Gomez J, Alektiar K, Hensley M, Soslow R, et al A nomogram for predicting overall survival of women with endometrial cancer following primary therapy: toward improving individualized cancer care. Gynecologic oncology. 2010;116(3):399–403. 10.1016/j.ygyno.2009.11.027 20022094 PMC3870336

[17] Polterauer S, Grimm C, Hofstetter G, Concin N, Natter C, Sturdza A, et al Nomogram prediction for overall survival of patients diagnosed with cervical cancer. British journal of cancer. 2012;107(6):918–24. 10.1038/bjc.2012.340 22871885 PMC3464766

[18] Liang W, Zhang L, Jiang G, Wang Q, Liu L, Liu D, et al Development and Validation of a Nomogram for Predicting Survival in Patients With Resected Non–Small-Cell Lung Cancer. Journal of Clinical Oncology. 2015:JCO. 2014.56. 6661. 10.1200/JCO.2014.56.6661 25624438

[19] Montero PH, Yu C, Palmer FL, Patel PD, Ganly I, Shah JP, et al Nomograms for preoperative prediction of prognosis in patients with oral cavity squamous cell carcinoma. Cancer. 2014;120(2):214–21. 10.1002/cncr.28407 . 24399417

[20] Ali S, Palmer FL, Yu C, DiLorenzo M, Shah JP, Kattan MW, et al A predictive nomogram for recurrence of carcinoma of the major salivary glands. JAMA Otolaryngol Head Neck Surg. 2013;139(7):698–705. 10.1001/jamaoto.2013.3347 . 23788168

[21] Velazquez ER, Hoebers F, Aerts HJ, Rietbergen MM, Brakenhoff RH, Leemans RC, et al Externally validated HPV-based prognostic nomogram for oropharyngeal carcinoma patients yields more accurate predictions than TNM staging. Radiotherapy and Oncology. 2014;113(3):324–30. 10.1016/j.radonc.2014.09.005 25443497

[22] Iasonos A, Schrag D, Raj GV, Panageas KS. How to build and interpret a nomogram for cancer prognosis. Journal of clinical oncology: official journal of the American Society of Clinical Oncology. 2008;26(8):1364–70. 10.1200/JCO.2007.12.9791 . 18323559

[23] Harrell FE Jr., Lee KL, Mark DB. Multivariable prognostic models: issues in developing models, evaluating assumptions and adequacy, and measuring and reducing errors. Stat Med. 1996;15(4):361–87. 10.1002/(SICI)1097-0258(19960229)15:4<361::AID-SIM168>3.0.CO;2-4 . 8668867

[24] Greiner M, Pfeiffer D, Smith R. Principles and practical application of the receiver-operating characteristic analysis for diagnostic tests. Preventive veterinary medicine. 2000;45(1):23–41. 10802332 10.1016/s0167-5877(00)00115-x

[25] Edge SB, Compton CC. The American Joint Committee on Cancer: the 7th edition of the AJCC cancer staging manual and the future of TNM. Annals of surgical oncology. 2010;17(6):1471–4. 10.1245/s10434-010-0985-4 . 20180029

[26] Guo R, Sun Y, Yu XL, Yin WJ, Li WF, Chen YY, et al Is primary tumor volume still a prognostic factor in intensity modulated radiation therapy for nasopharyngeal carcinoma? Radiotherapy and oncology: journal of the European Society for Therapeutic Radiology and Oncology. 2012;104(3):294–9. 10.1016/j.radonc.2012.09.001 . 22998947

[27] Chen C, Fei Z, Pan J, Bai P, Chen L. Significance of primary tumor volume and T-stage on prognosis in nasopharyngeal carcinoma treated with intensity-modulated radiation therapy. Jpn J Clin Oncol. 2011;41(4):537–42. 10.1093/jjco/hyq242 . 21242183

[28] Lee CC, Huang TT, Lee MS, Hsiao SH, Lin HY, Su YC, et al Clinical application of tumor volume in advanced nasopharyngeal carcinoma to predict outcome. Radiat Oncol. 2010;5:20 10.1186/1748-717X-5-20 20222940 PMC2842277

[29] Wu Z, Zeng RF, Su Y, Gu MF, Huang SM. Prognostic significance of tumor volume in patients with nasopharyngeal carcinoma undergoing intensity-modulated radiation therapy. Head Neck. 2013;35(5):689–94. 10.1002/hed.23010 . 22715047

[30] Johnson CR, Thames HD, Huang DT, Schmidt-Ullrich RK. The tumor volume and clonogen number relationship: tumor control predictions based upon tumor volume estimates derived from computed tomography. International journal of radiation oncology, biology, physics. 1995;33(2):281–7. . 7673015 10.1016/0360-3016(95)00119-j

[31] Lee AW, Lau WH, Tung SY, Chua DT, Chappell R, Xu L, et al Preliminary results of a randomized study on therapeutic gain by concurrent chemotherapy for regionally-advanced nasopharyngeal carcinoma: NPC-9901 Trial by the Hong Kong Nasopharyngeal Cancer Study Group. Journal of clinical oncology: official journal of the American Society of Clinical Oncology. 2005;23(28):6966–75. 10.1200/JCO.2004.00.7542 . 16192584

[32] Wang Y, Zhang Y, Ma S. Racial differences in nasopharyngeal carcinoma in the United States. Cancer Epidemiol. 2013;37(6):793–802. 10.1016/j.canep.2013.08.008 24035238 PMC3851929

[33] Marks JE, Phillips JL, Menck HR. The National Cancer Data Base report on the relationship of race and national origin to the histology of nasopharyngeal carcinoma. Cancer. 1998;83(3):582–8. . 9690553 10.1002/(sici)1097-0142(19980801)83:3<582::aid-cncr29>3.0.co;2-r

[34] Yip TT, Ngan RK, Fong AH, Law SC. Application of circulating plasma/serum EBV DNA in the clinical management of nasopharyngeal carcinoma. Oral oncology. 2014;50(6):527–38. 10.1016/j.oraloncology.2013.12.011 . 24440146

